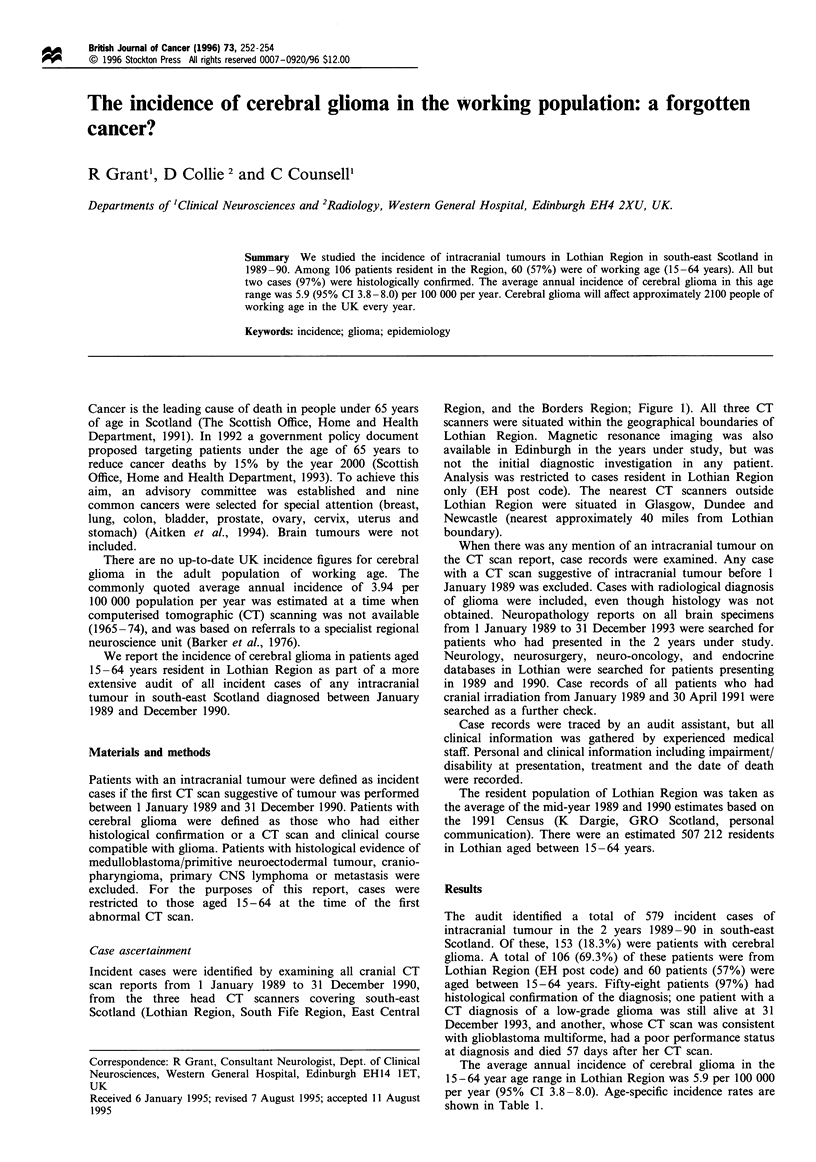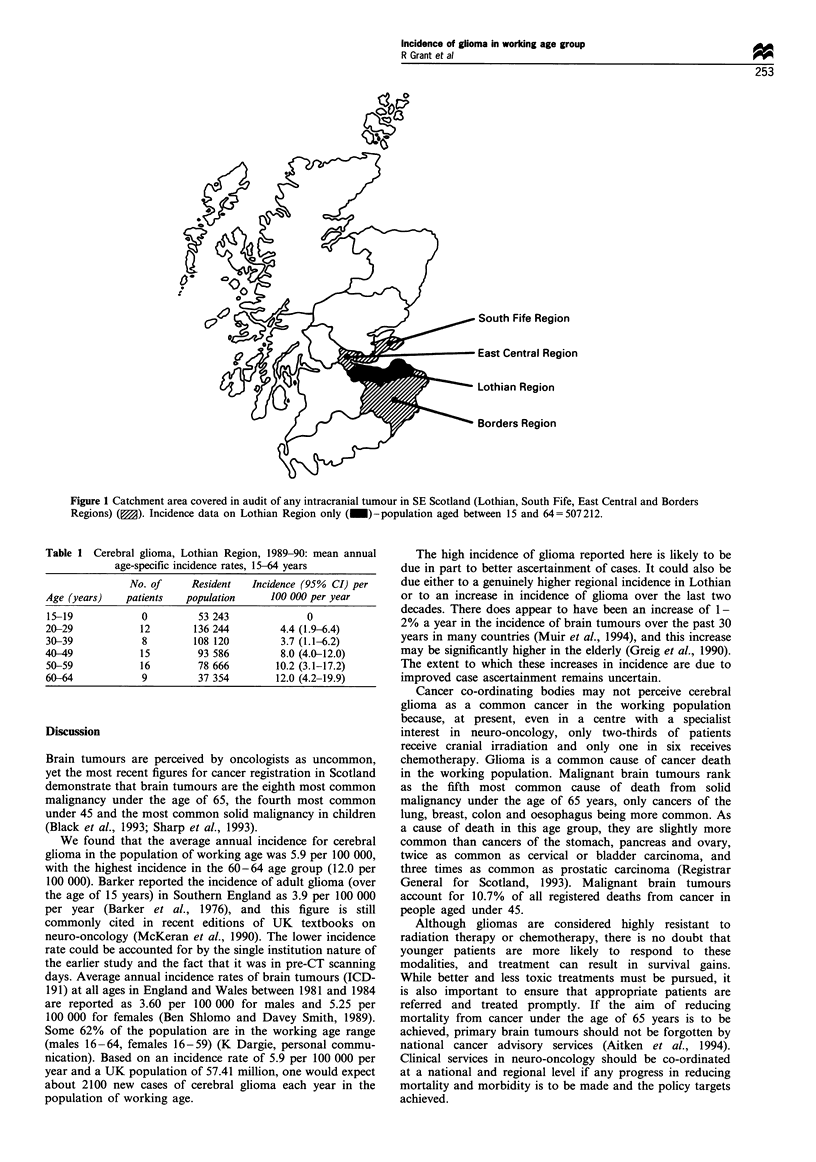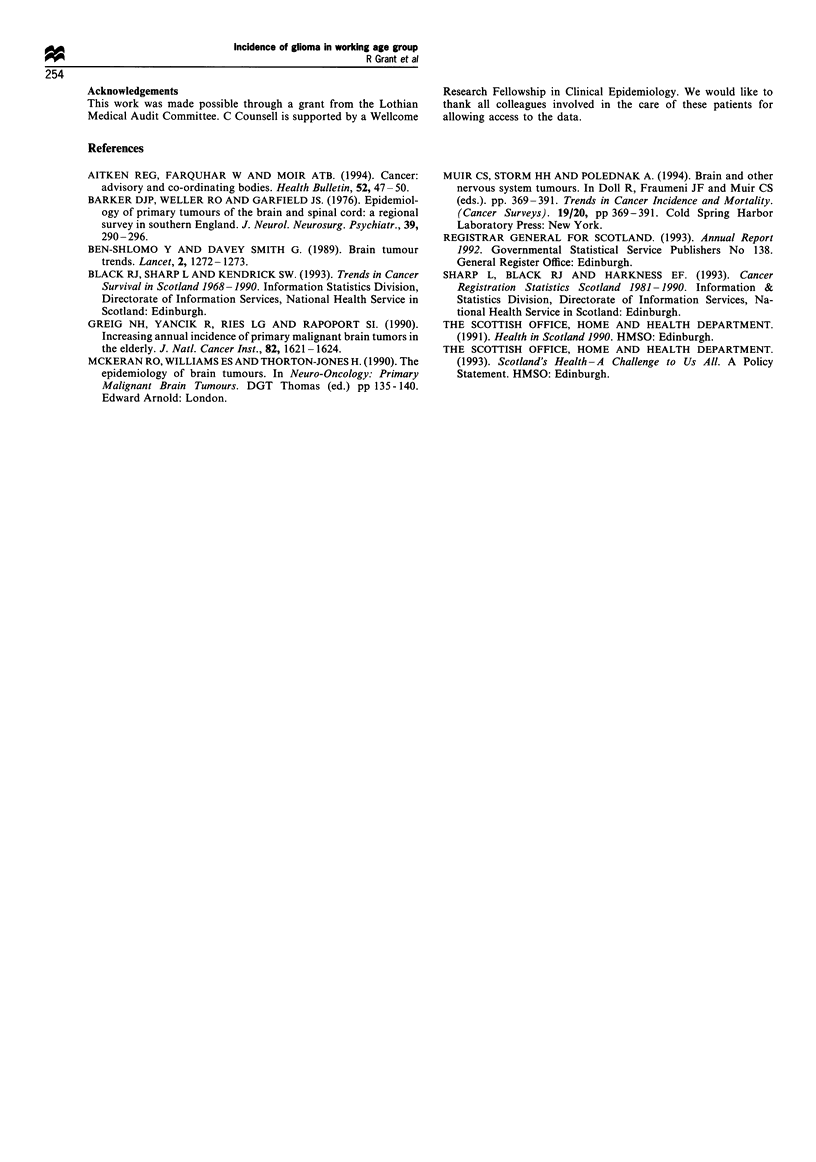# The incidence of cerebral glioma in the working population: a forgotten cancer?

**DOI:** 10.1038/bjc.1996.44

**Published:** 1996-01

**Authors:** R. Grant, D. Collie, C. Counsell

**Affiliations:** Department of Clinical Neurosciences, Western General Hospital, Edinburgh, UK.

## Abstract

We studied the incidence of intracranial tumours in Lothian Region in south-east Scotland in 1989-90. Among 106 patients resident in the Region, 60 (57%) were of working age (15-64 years). All but two cases (97%) were histologically confirmed. The average annual incidence of cerebral glioma in this age range was 5.9 (95% CI 3.8-8.0) per 100,000 per year. Cerebral glioma will affect approximately 2100 people of working age in the UK every year.


British Journal of Cancer (1996) 73, 252-254

© 1996 Stockton Press All rights reserved 0007-0920/96 $12.00

### The incidence of cerebral glioma in the working population: a forgotten cancer?

R Grant[1], D Collie [2] and C Counsell[1]

*Departments of [1]Clinical Neurosciences and [2]Radiology, Western General Hospital, Edinburgh EH4 2XU, UK.*

**Summary** We studied the incidence of intracranial tumours in Lothian Region in south-east Scotland in 1989-90. Among 106 patients resident in the Region, 60 (57%) were of working age (15-64 years). All but two cases (97%) were histologically confirmed. The average annual incidence of cerebral glioma in this age range was 5.9 (95% CI 3.8-8.0) per 100 000 per year. Cerebral glioma will affect approximately 2100 people of working age in the UK every year.

**Keywords:** incidence; glioma; epidemiology

Cancer is the leading cause of death in people under 65 years of age in Scotland (The Scottish Office, Home and Health Department, 1991). In 1992 a government policy document proposed targeting patients under the age of 65 years to reduce cancer deaths by 15% by the year 2000 (Scottish Office, Home and Health Department, 1993). To achieve this aim, an advisory committee was established and nine common cancers were selected for special attention (breast, lung, colon, bladder, prostate, ovary, cervix, uterus and stomach) (Aitken *et al.*, 1994). Brain tumours were not included.

There are no up-to-date UK incidence figures for cerebral glioma in the adult population of working age. The commonly quoted average annual incidence of 3.94 per 100 000 population per year was estimated at a time when computerised tomographic (CT) scanning was not available (1965-74), and was based on referrals to a specialist regional neuroscience unit (Barker *et al.*, 1976).

We report the incidence of cerebral glioma in patients aged 15-64 years resident in Lothian Region as part of a more extensive audit of all incident cases of any intracranial tumour in south-east Scotland diagnosed between January 1989 and December 1990.

### Materials and methods

Patients with an intracranial tumour were defined as incident cases if the first CT scan suggestive of tumour was performed between 1 January 1989 and 31 December 1990. Patients with cerebral glioma were defined as those who had either histological confirmation or a CT scan and clinical course compatible with glioma. Patients with histological evidence of medulloblastoma/primitive neuroectodermal tumour, craniopharyngioma, primary CNS lymphoma or metastasis were excluded. For the purposes of this report, cases were restricted to those aged 15-64 at the time of the first abnormal CT scan.

### *Case ascertainment*

Incident cases were identified by examining all cranial CT scan reports from 1 January 1989 to 31 December 1990, from the three head CT scanners covering south-east Scotland (Lothian Region, South Fife Region, East Central Region, and the Borders Region; Figure 1). All three CT scanners were situated within the geographical boundaries of Lothian Region. Magnetic resonance imaging was also available in Edinburgh in the years under study, but was not the initial diagnostic investigation in any patient. Analysis was restricted to cases resident in Lothian Region only (EH post code). The nearest CT scanners outside Lothian Region were situated in Glasgow, Dundee and Newcastle (nearest approximately 40 miles from Lothian boundary).

When there was any mention of an intracranial tumour on the CT scan report, case records were examined. Any case with a CT scan suggestive of intracranial tumour before 1 January 1989 was excluded. Cases with radiological diagnosis of glioma were included, even though histology was not obtained. Neuropathology reports on all brain specimens from 1 January 1989 to 31 December 1993 were searched for patients who had presented in the 2 years under study. Neurology, neurosurgery, neuro-oncology, and endocrine databases in Lothian were searched for patients presenting in 1989 and 1990. Case records of all patients who had cranial irradiation from January 1989 and 30 April 1991 were searched as a further check.

Case records were traced by an audit assistant, but all clinical information was gathered by experienced medical staff. Personal and clinical information including impairment/disability at presentation, treatment and the date of death were recorded.

The resident population of Lothian Region was taken as the average of the mid-year 1989 and 1990 estimates based on the 1991 Census (K Dargie, GRO Scotland, personal communication). There were an estimated 507 212 residents in Lothian aged between 15-64 years.

### Results

The audit identified a total of 579 incident cases of intracranial tumour in the 2 years 1989-90 in south-east Scotland. Of these, 153 (18.3%) were patients with cerebral glioma. A total of 106 (69.3%) of these patients were from Lothian Region (EH post code) and 60 patients (57%) were aged between 15-64 years. Fifty-eight patients (97%) had histological confirmation of the diagnosis; one patient with a CT diagnosis of a low-grade glioma was still alive at 31 December 1993, and another, whose CT scan was consistent with glioblastoma multiforme, had a poor performance status at diagnosis and died 57 days after her CT scan.

The average annual incidence of cerebral glioma in the 15-64 year age range in Lothian Region was 5.9 per 100 000 per year (95% CI 3.8-8.0). Age-specific incidence rates are shown in Table 1.

Correspondence: R Grant, Consultant Neurologist, Dept. of Clinical Neurosciences, Western General Hospital, Edinburgh EH14 1ET, UK
Received 6 January 1995; revised 7 August 1995; accepted 11 August 1995

**Figure 1** Catchment area covered in audit of any intracranial tumour in SE Scotland (Lothian, South Fife, East Central and Borders Regions) (▨). Incidence data on Lothian Region only (■)−population aged between 15 and 64 = 507 212.

**Table 1** Cerebral glioma, Lothian Region, 1989–90: mean annual age-specific incidence rates, 15–64 years

| *Age (years)* | *No. of patients* | *Resident population* | *Incidence (95% CI) per 100 000 per year* |
|---|---|---|---|
| 15–19 | 0 | 53 243 | 0 |
| 20–29 | 12 | 136 244 | 4.4 (1.9–6.4) |
| 30–39 | 8 | 108 120 | 3.7 (1.1–6.2) |
| 40–49 | 15 | 93 586 | 8.0 (4.0–12.0) |
| 50–59 | 16 | 78 666 | 10.2 (3.1–17.2) |
| 60–64 | 9 | 37 354 | 12.0 (4.2–19.9) |

### Discussion

Brain tumours are perceived by oncologists as uncommon, yet the most recent figures for cancer registration in Scotland demonstrate that brain tumours are the eighth most common malignancy under the age of 65, the fourth most common under 45 and the most common solid malignancy in children (Black *et al.*, 1993; Sharp *et al.*, 1993).

We found that the average annual incidence for cerebral glioma in the population of working age was 5.9 per 100 000, with the highest incidence in the 60–64 age group (12.0 per 100 000). Barker reported the incidence of adult glioma (over the age of 15 years) in Southern England as 3.9 per 100 000 per year (Barker *et al.*, 1976), and this figure is still commonly cited in recent editions of UK textbooks on neuro-oncology (McKeran *et al.*, 1990). The lower incidence rate could be accounted for by the single institution nature of the earlier study and the fact that it was in pre-CT scanning days. Average annual incidence rates of brain tumours (ICD-191) at all ages in England and Wales between 1981 and 1984 are reported as 3.60 per 100 000 for males and 5.25 per 100 000 for females (Ben Shlomo and Davey Smith, 1989). Some 62% of the population are in the working age range (males 16–64, females 16–59) (K Dargie, personal communication). Based on an incidence rate of 5.9 per 100 000 per year and a UK population of 57.41 million, one would expect about 2100 new cases of cerebral glioma each year in the population of working age.

The high incidence of glioma reported here is likely to be due in part to better ascertainment of cases. It could also be due either to a genuinely higher regional incidence in Lothian or to an increase in incidence of glioma over the last two decades. There does appear to have been an increase of 1–2% a year in the incidence of brain tumours over the past 30 years in many countries (Muir *et al.*, 1994), and this increase may be significantly higher in the elderly (Greig *et al.*, 1990). The extent to which these increases in incidence are due to improved case ascertainment remains uncertain.

Cancer co-ordinating bodies may not perceive cerebral glioma as a common cancer in the working population because, at present, even in a centre with a specialist interest in neuro-oncology, only two-thirds of patients receive cranial irradiation and only one in six receives chemotherapy. Glioma is a common cause of cancer death in the working population. Malignant brain tumours rank as the fifth most common cause of death from solid malignancy under the age of 65 years, only cancers of the lung, breast, colon and oesophagus being more common. As a cause of death in this age group, they are slightly more common than cancers of the stomach, pancreas and ovary, twice as common as cervical or bladder carcinoma, and three times as common as prostatic carcinoma (Registrar General for Scotland, 1993). Malignant brain tumours account for 10.7% of all registered deaths from cancer in people aged under 45.

Although gliomas are considered highly resistant to radiation therapy or chemotherapy, there is no doubt that younger patients are more likely to respond to these modalities, and treatment can result in survival gains. While better and less toxic treatments must be pursued, it is also important to ensure that appropriate patients are referred and treated promptly. If the aim of reducing mortality from cancer under the age of 65 years is to be achieved, primary brain tumours should not be forgotten by national cancer advisory services (Aitken *et al.*, 1994). Clinical services in neuro-oncology should be co-ordinated at a national and regional level if any progress in reducing mortality and morbidity is to be made and the policy targets achieved.

**Acknowledgements**
This work was made possible through a grant from the Lothian Medical Audit Committee. C Counsell is supported by a Wellcome Research Fellowship in Clinical Epidemiology. We would like to thank all colleagues involved in the care of these patients for allowing access to the data.

### References

AITKEN REG, FARQUHAR W AND MOIR ATB. (1994). Cancer: advisory and co-ordinating bodies. *Health Bulletin*, **52**, 47–50.

BARKER DJP, WELLER RO AND GARFIELD JS. (1976). Epidemiology of primary tumours of the brain and spinal cord: a regional survey in southern England. *J. Neurol. Neurosurg. Psychiatr.*, **39**, 290–296.

BEN-SHLOMO Y AND DAVEY SMITH G. (1989). Brain tumour trends. *Lancet*, **2**, 1272–1273.

BLACK RJ, SHARP L AND KENDRICK SW. (1993). *Trends in Cancer Survival in Scotland 1968–1990*. Information Statistics Division, Directorate of Information Services, National Health Service in Scotland: Edinburgh.

GREIG NH, YANCIK R, RIES LG AND RAPOPORT SI. (1990). Increasing annual incidence of primary malignant brain tumors in the elderly. *J. Natl. Cancer Inst.*, **82**, 1621–1624.

MCKERAN RO, WILLIAMS ES AND THORTON-JONES H. (1990). The epidemiology of brain tumours. In *Neuro-Oncology: Primary Malignant Brain Tumours*. DGT Thomas (ed.) pp 135-140. Edward Arnold: London.

MUIR CS, STORM HH AND POLEDNAK A. (1994). Brain and other nervous system tumours. In Doll R, Fraumeni JF and Muir CS (eds.). pp. 369–391. *Trends in Cancer Incidence and Mortality. (Cancer Surveys).* **19/20,** pp 369–391. Cold Spring Harbor Laboratory Press: New York.

REGISTRAR GENERAL FOR SCOTLAND. (1993). *Annual Report 1992.* Governmental Statistical Service Publishers No 138. General Register Office: Edinburgh.

SHARP L, BLACK RJ AND HARKNESS EF. (1993). *Cancer Registration Statistics Scotland 1981–1990.* Information & Statistics Division, Directorate of Information Services, National Health Service in Scotland: Edinburgh.

THE SCOTTISH OFFICE, HOME AND HEALTH DEPARTMENT. (1991). *Health in Scotland 1990.* HMSO: Edinburgh.

THE SCOTTISH OFFICE, HOME AND HEALTH DEPARTMENT. (1993). *Scotland's Health–A Challenge to Us All.* A Policy Statement. HMSO: Edinburgh.